# Supplementary material for: High-resolution, high-throughput detection of hidden antibiotic resistance with the dilution-and-delay (DnD) susceptibility assay
Source: Nat Commun. 2026 Mar 7;17:3641. doi: 10.1038/s41467-026-70174-z (PMC13096344; doi:10.1038/s41467-026-70174-z)
Supplement: Supplementary file 1 — Supplementary Information [file 41467_2026_70174_MOESM1_ESM.pdf]

Supplementary Information

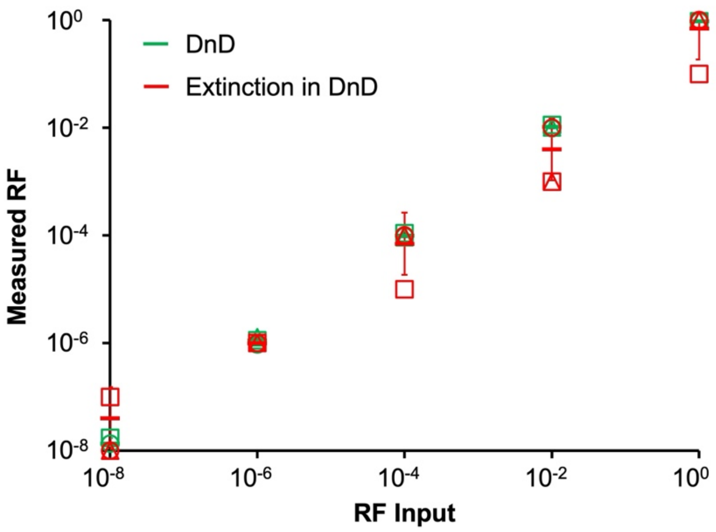

Supplementary Fig. 1. Validation of the Dilution-and-Delay (DnD) assay using defined input mixtures.

As described in the main text, a colistin-susceptible strain (AMK105) was mixed with a fully resistant strain (AMK104) at defined input frequencies. Resistance frequencies (RFs) were measured. Green symbols indicate RFs measured from the wells showing growth recovery (adopted from Fig. 1f). Red symbols indicate RFs from extinction points. Their RFs were within a 10-fold range, confirming the internal consistency. Each experiment was performed in biological triplicate. Individual replicates are shown as different symbols, with lines and error bars indicating the mean and standard deviation.

a

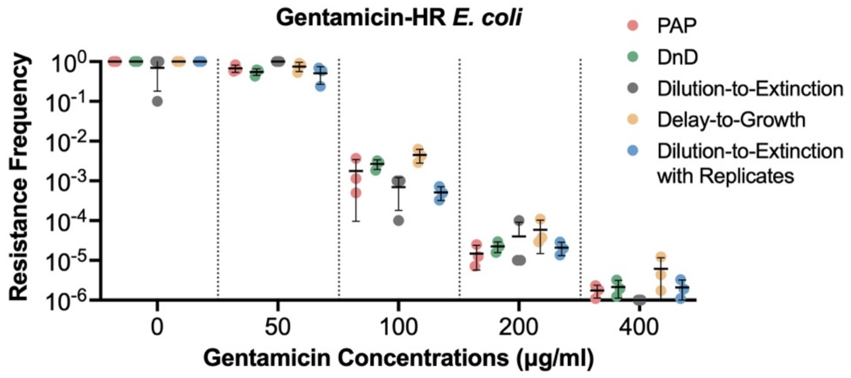

b

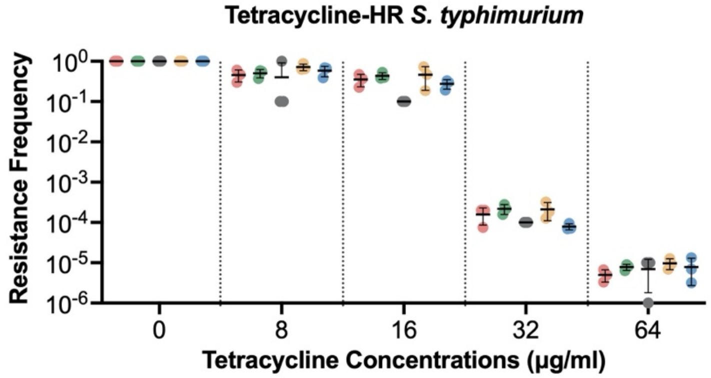

Supplementary Fig. 2: Validation of the DnD assay against heteroresistant clinical isolates.

a–b. In addition to the three heteroresistant (HR) strains shown in Fig. 2, resistance frequencies (RF) were measured for two additional clinical HR isolates: gentamicin-HR *E. coli* (AMK118) and tetracycline-HR *S. typhimurium* (AMK120). Each experiment was performed in biological triplicate. Individual replicates are shown as different symbols, with black lines and error bars indicating the mean and standard deviation. All three assays recapitulated PAP-derived RF, RF_{pap}, across antibiotics and concentrations.

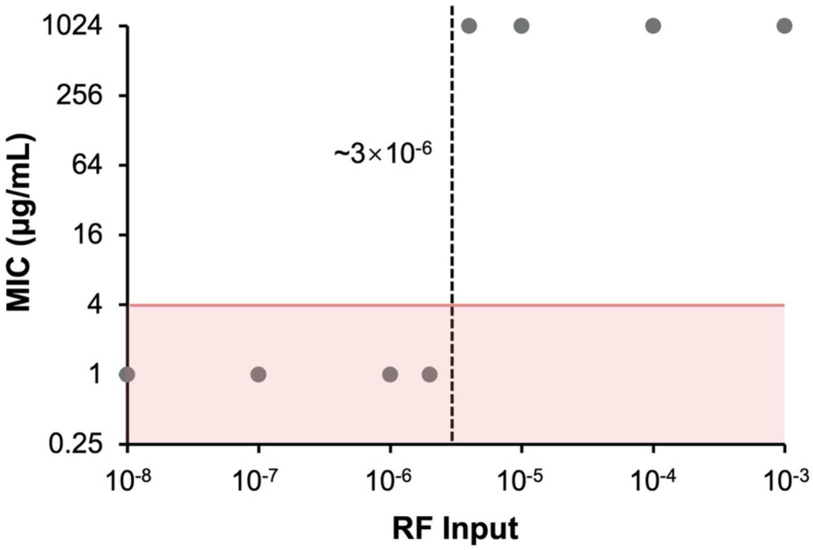

Supplementary Fig. 3: MIC of artificial mixtures containing defined ratios of fully susceptible and fully resistant cells.

The MIC of mixtures containing defined ratios of colistin-susceptible (AMK105) and resistant (AMK104) *E. cloacae* cells was determined by a visual assessment of growth in wells inoculated with a standard inoculum size (~5×10^5 cells). A sharp transition occurred when resistant frequency (RF) input exceeded ~3×10^{-6} (vertical dashed line), shifting the classification from "susceptible" (below breakpoint, horizontal red line) to "resistant" (above breakpoint).

a

Strain	Species	Antibiotic Tested	MIC (μg/mL)
AMK107	*E. cloacae*	Colistin	512
AMK117	*E. coli*	Tobramycin	40
AMK118	*E.coli*	Gentamicin	400
AMK120	*S. typhimurium*	Tetracycline	64
KMK4	*K. pneumoniae*	Meropenem	32

b

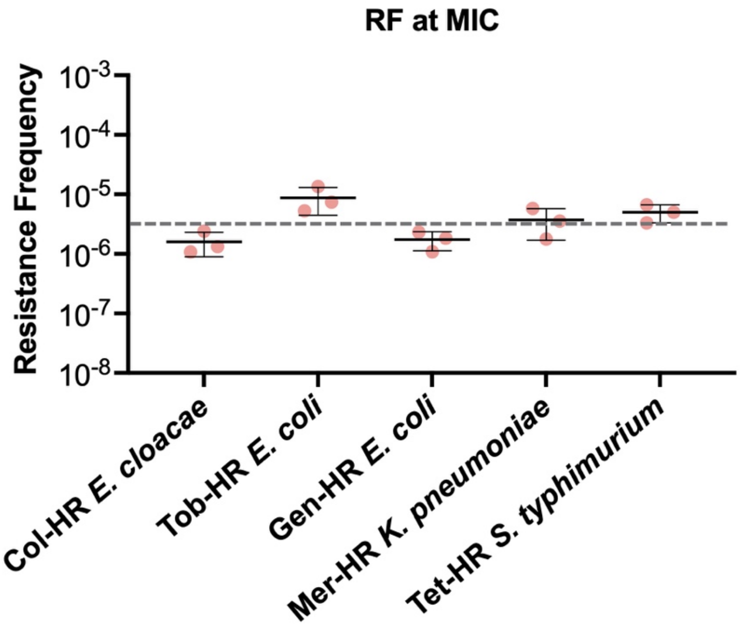

Supplementary Fig. 4: Resistance frequency (RF) of endogenously heteroresistant (HR) strains at the MIC of antibiotics.

a. MIC of previously characterized HR strains (Fig. 2 and Supplementary Fig. 2) was measured by visually inspecting the growth in wells inoculated with the standard inoculum size.

b. RF at their respective MIC value was plotted for each strain. The dashed line indicates RF = 3×10^{-6}. Each experiment was performed in biological triplicate. Black lines and error bars indicating the mean and standard deviation.

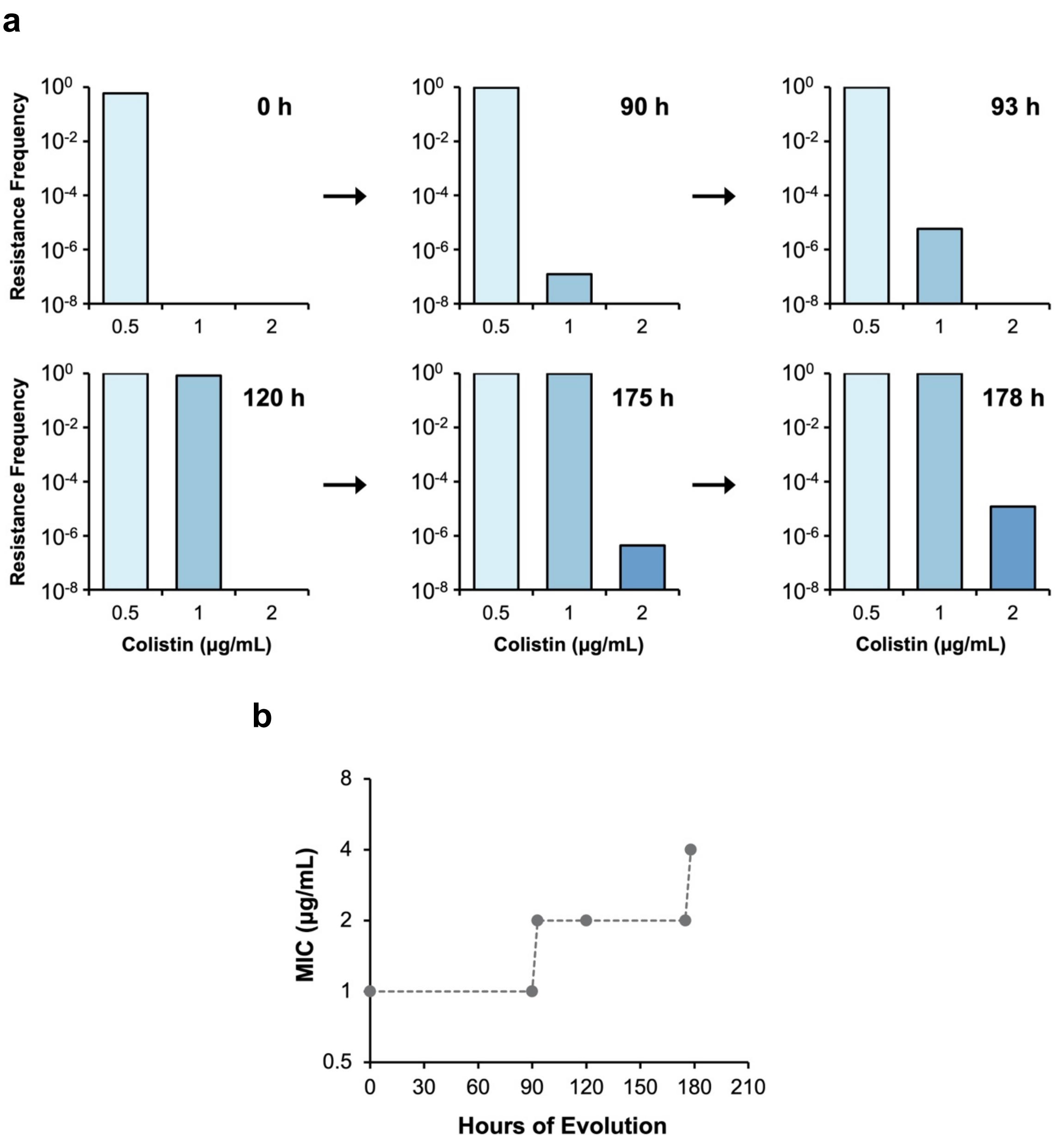

Supplementary Fig. 5: Evolutionary dynamics in a susceptible *E. cloacae* strain under colistin exposure.

a-b. Colistin-susceptible *E. cloacae* cells (AMK105) were continuously exposed to colistin for resistance evolution. Resistance frequency was monitored over time using DnD, identifying the emergences of mutants at 90th and 175th hour.

b) MIC changes of the evolved strain determined by standard criteria.

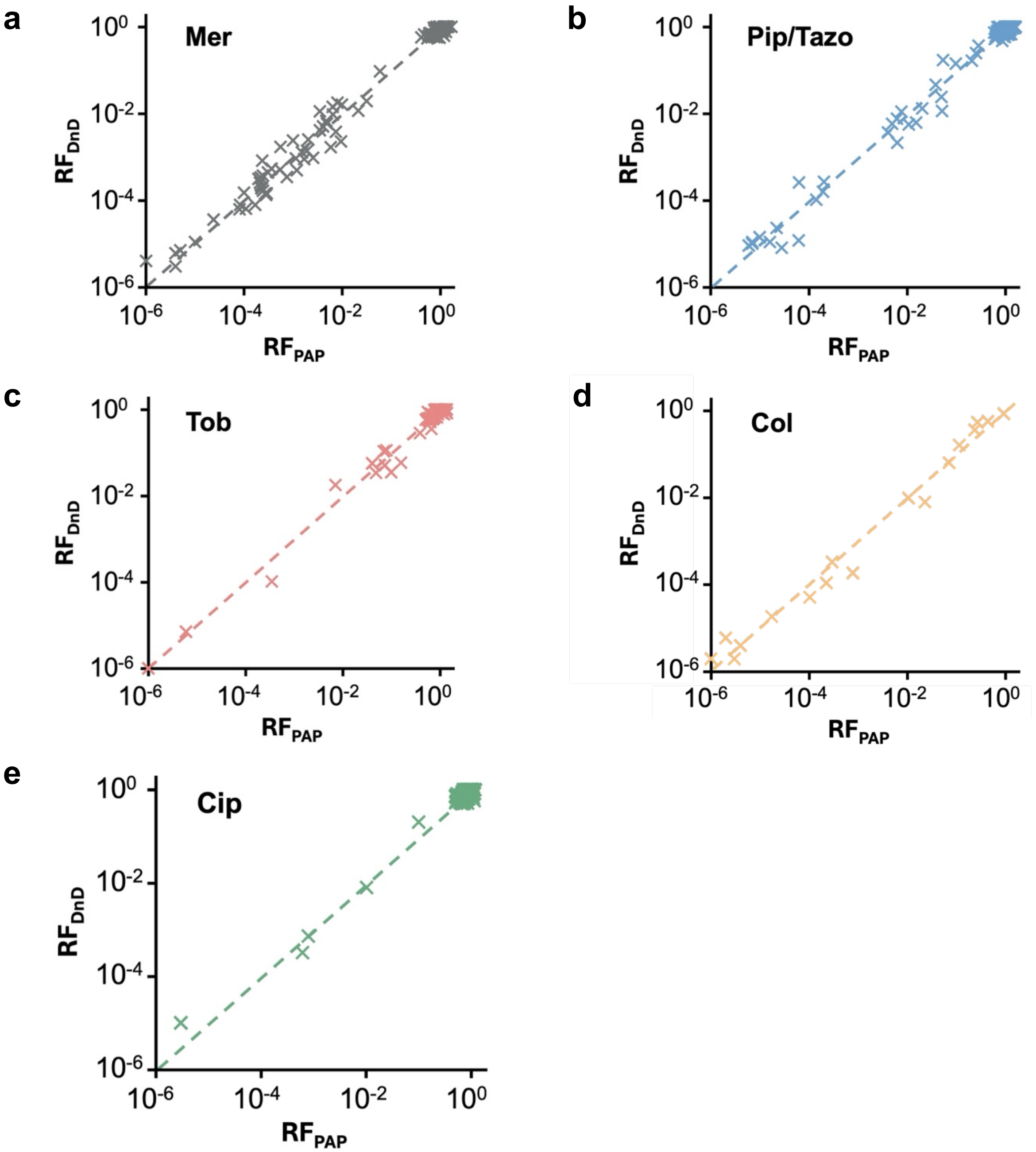

Supplementary Fig. 6: Comparing resistance frequency (RF) measured using the DnD (RF_{DnD}) and PAP (RF_{PAP}) across clinical isolates.

a-e. RF_{DnD} measured in Table 1 (n = 118) were plotted against RF_{PAP} for each antibiotic: a) meropenem (Mer), b) piperacillin/tazobactam (Pip/Tazo), c) tobramycin (Tob), d) colistin (Col), and e) ciprofloxacin (Cip). The diagonal dashed line denotes y = x (the line of equality).

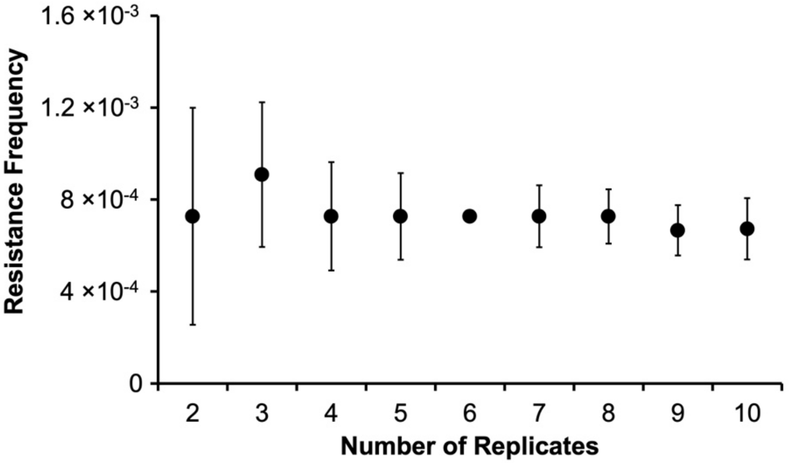

Supplementary Fig. 7. Increasing the number of replicates improves the precision of dilution-to-extinction assays.

Resistant frequencies (RF) were calculated using most probable number (MPN) analysis applied to dilution-to-extinction data with varying numbers of biological replicates. Increasing the number of replicates reduced the uncertainty (as reflected by narrower confidence intervals), indicating the use of MPN-based analysis with a moderate number of replicates (e.g., $n \geq 5$) as a practical approach to improve RF quantification. Black lines and error bars indicating the mean and standard deviation.

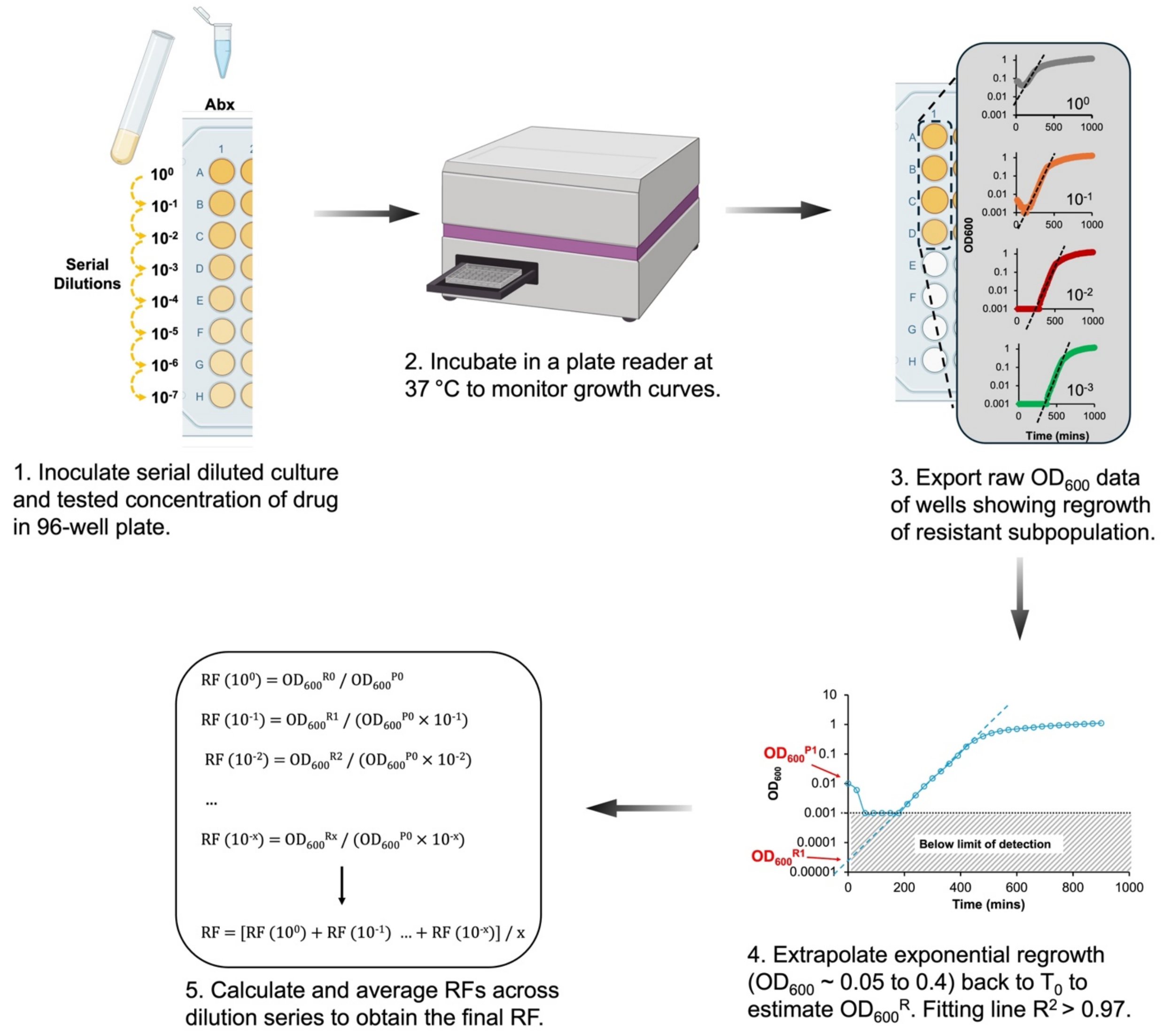

Supplementary Fig. 8. Workflow of the DnD assay and data analysis pipeline.

Serially diluted cultures are inoculated into a 96-well plate containing the tested antibiotic and incubated at 37 °C with continuous OD_{600} monitoring (1–2). Raw OD_{600} growth curves from wells showing delayed regrowth are exported (3). Exponential regrowth phases ($OD_{600} \approx 0.05$–0.4) are back-extrapolated to time zero to estimate the initial OD_{600} contributed by resistant cells, retaining only fits with $R^2 > 0.97$ (4). Resistance frequencies are calculated for each dilution relative to the undiluted control and averaged across the dilution series to obtain the final RF estimate (5). See the corresponding tab in the Source Data file for quantitative analysis. Created in BioRender. Ma, M. (2026) https://BioRender.com/i24dnsu

Supplementary Table 1. High-throughput Screening of ~120 clinical isolates. We determined their resistance frequency (RF); see Source data. Our main text justifies three distinct categorizations: resistant (R, RF > 0.5), heteroresistant (HR, 10^{-7} < RF < 0.5), or susceptible (S, RF < 10^{-7}). Out of 590 classifications, DnD and PAP matched in all but four cases, corresponding to a 99.3 % agreement rate. The few discrepancies (highlighted in red) resulted from minor differences in RF values near classification thresholds. For instance, the strain KMK70 treated with colistin showed an RF of 58.2 % by DnD and 43.9% by PAP—both indicating high resistance, though falling on opposite sides of the 50% threshold. These mismatches reflect the use of artificial cutoff values, which can vary between studies [1-4], rather than substantive discrepancy between DnD and PAP tests.

		Mer		Pip/Tazo		Tob		Col		Cip				Mer		Pip/Tazo		Tob		Col		Cip	
**KMK#**	**Species**	**PAP**	**DnD**	**PAP**	**DnD**	**PAP**	**DnD**	**PAP**	**DnD**	**PAP**	**DnD**	**KMK #**	**Species**	**PAP**	**DnD**	**PAP**	**DnD**	**PAP**	**DnD**	**PAP**	**DnD**	**PAP**	**DnD**
25	*E. coli*	S	S	HR	HR	S	S	S	S	R	R	54	*K. p.*	R	R	R	R	S	S	S	S	R	R
28	*E. coli*	HR	HR	HR	HR	HR	HR	S	S	R	R	55	*K. p.*	R	R	HR	HR	S	S	S	S	R	R
29	*E. coli*	HR	HR	R	R	R	R	S	S	R	R	57	*K. p.*	HR	HR	R	R	HR	HR	S	S	R	R
35	*E. coli*	HR	HR	HR	HR	R	R	S	S	R	R	58	*K. p.*	R	R	R	R	HR	HR	S	S	R	R
44	*E. coli*	HR	HR	HR	HR	HR	HR	S	S	HR	HR	59	*K. p.*	HR	HR	R	R	R	R	S	S	R	R
60	*E. coli*	HR	HR	HR	HR	R	R	S	S	R	R	61	*K. p.*	HR	HR	R	R	R	R	S	S	R	R
85	*E. coli*	HR	HR	HR	HR	R	R	S	S	R	R	62	*K. p.*	R	R	R	R	R	R	S	S	R	R
88	*E. coli*	HR	HR	R	R	S	S	S	S	R	R	63	*K. p.*	R	R	R	R	S	S	S	S	R	R
97	*E. coli*	HR	HR	HR	HR	S	S	S	S	S	S	64	*K. p.*	R	R	R	R	S	S	S	S	R	R
99	*E. coli*	S	S	HR	HR	S	S	S	S	R	R	65	*K. p.*	R	R	R	R	S	S	S	S	R	R
7	*E. c.*	HR	HR	S	S	S	S	S	S	R	R	66	*K. p.*	HR	HR	R	R	S	S	S	S	S	S
22	*E. c.*	HR	HR	R	R	S	S	S	S	R	R	67	*K. p.*	R	R	R	R	S	S	S	S	R	R
53	*E. c.*	R	R	R	R	R	R	HR	HR	R	R	68	*K. p.*	R	R	HR	HR	R	R	S	S	R	R
56	*E. c.*	S	S	S	S	S	S	HR	HR	HR	HR	69	*K. p.*	R	R	HR	HR	R	R	S	S	R	R
75	*E. c.*	S	S	S	S	S	S	HR	HR	R	R	70	*K. p.*	HR	HR	HR	HR	R	R	HR	R	R	R
81	*E. c.*	HR	HR	HR	HR	R	R	S	S	R	R	71	*K. p.*	R	R	R	R	R	R	S	S	R	R
82	*E. c.*	HR	HR	HR	HR	R	R	S	S	R	R	72	*K. p.*	R	R	R	R	R	R	S	S	R	R
4	*K. p.*	HR	HR	R	R	R	R	S	S	R	R	73	*K. p.*	R	R	R	R	R	R	HR	HR	R	R
5	*K. p.*	R	R	R	R	S	S	S	S	R	R	74	*K. p.*	R	R	R	R	R	R	S	S	R	R
6	*K. p.*	R	R	R	R	S	S	S	S	R	R	76	*K. p.*	S	S	S	S	R	R	S	S	R	R
8	*K. p.*	HR	HR	R	R	S	S	S	S	R	R	77	*K. p.*	HR	HR	R	R	R	R	S	S	R	R
9	*K. p.*	HR	HR	R	R	HR	HR	S	S	R	R	78	*K. p.*	R	R	HR	HR	R	R	HR	R	R	R
10	*K. p.*	HR	HR	HR	HR	S	S	S	S	R	R	79	*K. p.*	HR	HR	HR	HR	R	R	S	S	R	R
11	*K. p.*	HR	HR	R	R	S	S	S	S	R	R	80	*K. p.*	HR	HR	HR	HR	R	R	S	S	R	R
12	*K. p.*	HR	HR	R	R	S	S	S	S	R	R	83	*K. p.*	S	S	S	S	R	R	HR	HR	R	R
13	*K. p.*	HR	HR	R	R	HR	HR	S	S	HR	HR	84	*K. p.*	HR	HR	HR	HR	R	R	S	S	R	R
14	*K. p.*	R	R	R	R	S	S	S	S	HR	HR	86	*K. p.*	HR	HR	R	R	R	R	S	S	R	R
15	*K. p.*	HR	HR	R	R	HR	HR	S	S	R	R	87	*K. p.*	R	R	R	R	R	R	S	S	R	R
16	*K. p.*	HR	HR	R	R	HR	HR	S	S	R	R	89	*K. p.*	R	R	HR	HR	S	S	HR	HR	R	R
17	*K. p.*	HR	HR	R	R	S	S	S	S	R	R	90	*K. p.*	R	R	HR	HR	S	S	S	S	R	R
18	*K. p.*	R	R	R	R	S	S	S	S	R	R	91	*K. p.*	R	R	R	R	R	R	S	S	R	R
19	*K. p.*	R	R	R	R	S	S	S	S	R	R	92	*K. p.*	HR	HR	R	R	R	R	S	S	R	R
20	*K. p.*	R	R	R	R	HR	HR	S	S	R	R	93	*K. p.*	R	R	R	R	R	R	S	S	R	R
21	*K. p.*	HR	HR	R	R	S	S	S	S	S	S	94	*K. p.*	HR	HR	HR	HR	R	R	S	S	R	R
23	*K. p.*	R	R	R	R	R	R	HR	HR	R	R	95	*K. p.*	R	R	R	R	R	R	HR	HR	R	R
24	*K. p.*	R	R	R	R	S	S	S	S	R	R	96	*K. p.*	R	R	R	R	R	R	S	S	R	R
26	*K. p.*	HR	HR	HR	HR	S	S	S	S	R	R	98	*K. p.*	R	R	R	R	R	R	S	S	R	R
27	*K. p.*	R	R	R	R	S	S	S	S	R	R	99	*P. a.*	R	R	R	R	S	S	S	S	S	S
30	*K. p.*	R	R	R	R	S	S	HR	HR	R	R	100	*P. a.*	HR	HR	HR	HR	S	S	HR	HR	S	S
31	*K. p.*	HR	R	HR	HR	R	R	HR	HR	R	R	101	*P. a.*	R	R	R	R	S	S	S	S	S	S
32	*K. p.*	R	R	R	R	HR	HR	S	S	R	R	102	*P. a..*	R	R	R	R	S	S	S	S	S	S
33	*K. p.*	HR	HR	HR	HR	R	R	HR	HR	R	R	103	*P. a.*	R	R	R	R	S	S	S	S	S	S
34	*K. p.*	R	R	R	R	S	S	HR	HR	R	R	104	*P. a.*	HR	HR	HR	HR	HR	HR	S	S	HR	HR
36	*K. p.*	R	R	R	R	S	S	S	S	R	R	105	*P. a.*	HR	HR	HR	HR	S	S	S	S	S	S
37	*K. p.*	HR	HR	R	R	R	R	HR	HR	R	R	106	*P. a.*	HR	HR	HR	HR	S	S	S	S	S	S
38	*K. p.*	HR	HR	R	R	S	S	S	S	R	R	107	*P. a.*	R	R	R	R	S	S	S	S	S	S
39	*K. p.*	R	R	R	R	R	R	S	S	R	R	108	*P. a.*	R	R	R	R	S	S	S	S	S	S
40	*K. p.*	HR	HR	R	R	HR	HR	S	S	R	R	109	*P. a.*	R	R	R	R	S	S	S	S	S	S
41	*K. p.*	HR	HR	R	R	S	S	S	S	R	R	110	*P. a.*	HR	R	R	R	S	S	S	S	S	S
42	*K. p.*	HR	HR	HR	HR	S	S	HR	HR	R	R	111	*A. b.*	R	R	R	R	R	R	S	S	S	S
43	*K. p.*	R	R	R	R	S	S	S	S	R	R	112	*A. b.*	R	R	R	R	S	S	S	S	R	R
45	*K. p.*	HR	HR	R	R	S	S	S	S	S	S	113	*A. b.*	R	R	R	R	R	R	R	R	R	R
46	*K. p.*	HR	HR	R	R	S	S	S	S	R	R	114	*A. b.*	R	R	R	R	R	R	S	S	R	R
47	*K. p.*	HR	HR	R	R	S	S	S	S	R	R	115	*A. b.*	R	R	R	R	R	R	S	S	S	S
48	*K. p.*	HR	HR	HR	HR	S	S	S	S	R	R	116	*A. b.*	R	R	R	R	S	S	S	S	S	S
49	*K. p.*	R	R	R	R	S	S	S	S	R	R	117	*A. b.*	R	R	R	R	S	S	S	S	R	R
50	*K. p.*	R	R	R	R	S	S	S	S	R	R	118	*A. b.*	R	R	R	R	R	R	HR	HR	R	R
51	*K. p.*	HR	HR	R	R	S	S	S	S	R	R	119	*A. b.*	R	R	R	R	R	R	S	S	R	R
52	*K. p.*	HR	HR	R	R	S	S	S	S	R	R	120	*A. b.*	R	R	R	R	R	R	HR	HR	R	R

Note: *K.p*: *Klebsiella pneumoniae*, *E. coli*: *Escherichia coli*, *E. c*: *Enterobacter cloacae, P. a*: *Pseudomonas aeruginosa, A. b*: *Acinetobacter baumannii*. Mer: meropenem, Pip/Tazo: piperacillin, Tob: tobramycin, Col: colistin, Cip: ciprofloxacin.

Supplementary References.

1 Band, V. I. *et al.* Antibiotic failure mediated by a resistant subpopulation in Enterobacter cloacae. *Nature Microbiology* **1**, 16053 (2016). https://doi.org/10.1038/nmicrobiol.2016.53

2 Pereira, C., Larsson, J., Hjort, K., Elf, J. & Andersson, D. I. The highly dynamic nature of bacterial heteroresistance impairs its clinical detection. *Communications Biology* **4**, 521 (2021). https://doi.org/10.1038/s42003-021-02052-x

3 Andersson, D. I., Nicoloff, H. & Hjort, K. Mechanisms and clinical relevance of bacterial heteroresistance. *Nature Reviews Microbiology* **17**, 479-496 (2019). https://doi.org/10.1038/s41579-019-0218-1

4 El-Halfawy, O. M. & Valvano, M. A. Antimicrobial Heteroresistance: an Emerging Field in Need of Clarity. *Clinical Microbiology Reviews* **28**, 191-207 (2015). https://doi.org/10.1128/cmr.00058-14
